# Ectoparasites of the critically endangered insular cavy, *Cavia intermedia* (Rodentia: Caviidae), southern Brazil

**DOI:** 10.1016/j.ijppaw.2014.12.009

**Published:** 2015-01-07

**Authors:** André Luis Regolin, Nina Furnari, Fernando de Castro Jacinavicius, Pedro Marcos Linardi, Carlos José de Carvalho-Pinto

**Affiliations:** a Departamento de Biologia, Universidade Federal de Santa Maria, Santa Maria 97110-970, Brazil; b Departamento de Psicologia Experimental, Universidade de São Paulo, São Paulo 05508-030, Brazil; c Instituto Butantan, São Paulo 05503900, Brazil; d Departamento de Parasitologia, Universidade Federal de Minas Gerais, Belo Horizonte 31270-901, Brazil; e Departamento de Microbiologia e Parasitologia, Universidade Federal de Santa Catarina, Florianópolis 88040-900, Brazil

**Keywords:** Chewing lice, Gyropidae, Insular syndrome, Island biogeography, Trimenoponidae, Trombiculidae

## Abstract

*Cavia intermedia* is a rodent species critically endangered and is found only on a 10 hectare island off the southern Brazilian coast. To identify the ectoparasites of *C. intermedia*, 27 specimens (14 males and 13 females), representing approximately 65% of the estimated total population, were captured and examined. A total of 1336 chewing lice of two species were collected: *Gliricola lindolphoi* (Amblycera: Gyropidae) and *Trimenopon hispidum* (Amblycera: Trimenoponidae). In addition, chiggers *Arisocerus hertigi* (Acari: Trombiculidae) and *Eutrombicula* sp. (Acari: Trombiculidae) were collected from the ears of all captured animals. This low species richness compared to those for other *Cavia* species is expected for island mammals. Although the results presented here are not conclusive about the relationship between *C. intermedia* and ectoparasites, this low species richness found might be reflected in a low level of investment by the hosts in the basal immune defense, since investments in white blood cell production by mammals are influenced by the diversity of parasites in the environment. Additionally, considering that it might result in host vulnerability to other parasites that might be introduced through exotic or migratory host species, the monitoring of *C. intermedia*, including parasitological and immunological assessments, is recommended as a key component of conservation efforts.

## Highlights

• The parasitological fauna associated at *Cavia intermedia* was previously unknown.
• We sampled ectoparasites from 65% of the total host population.
• The louse species *Gliricola lindolphoi* and *Trimenopon hispidum* were collected.
• The trombiculid mites *Arisocerus hertigi* and *Eutrombicula* sp. were also collected.
• We compared the results to ectoparasite records for other *Cavia* species.

## Graphical Abstract

**Figure:**
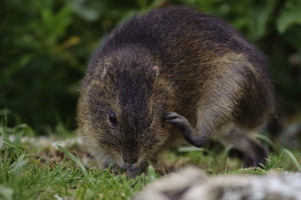

## 1 Introduction

Species richness on islands results from a dynamic balance between migration and extinction processes, which depend on island size and its distance from the adjacent continent (MacArthur and Wilson, 1967). Thus, insular environments are characterized primarily by a decrease in species richness. As a consequence, many biological processes are affected and differentiated from those on the mainland, including host–parasite interactions, mainly through differences in the number of parasite species, their biological characteristics and host specificity (Magnanou and Morand, 2006). Parasites play an important role in host ecology, immune investment, population dynamics, and behavior, so it seems relevant to identify and quantify the parasite assemblages associated with archipelago mammals, specially the insular endemic species (Linardi and Guimarães, 2000; Berglund et al., 2009; Bordes and Morand, 2009).

*Cavia intermedia* is an endemic species from the largest island (9.86 ha) of Moleques do Sul Archipelago, in Santa Catarina State, Southern Brazil (Cherem et al., 1999). The species probably diverged from a common ancestral population of *C. magna* as the result of vicariance associated with archipelago formation, approximately 8000 years ago (Gava et al., 1998; Cherem et al., 1999; Furnari, 2013). It is categorized as “critically endangered” at the global level according to the International Union for Conservation of Nature (IUCN) criteria (Chapman, 2008). It is probably the mammal with the smallest geographic distribution in the world (Alcover et al., 1998) and its average population size was estimated as just 42 individuals (Salvador and Fernandez, 2008). The ectoparasites of this species have not previously been described.

The aim of this study was to document the ectoparasites of *C. intermedia*, record their prevalence and abundance, examine the influence of host sex on these parameters and describe the host–parasite interactions based on comparisons to other species of the genus *Cavia*.

## 2 Methods

**Fig. 1:**
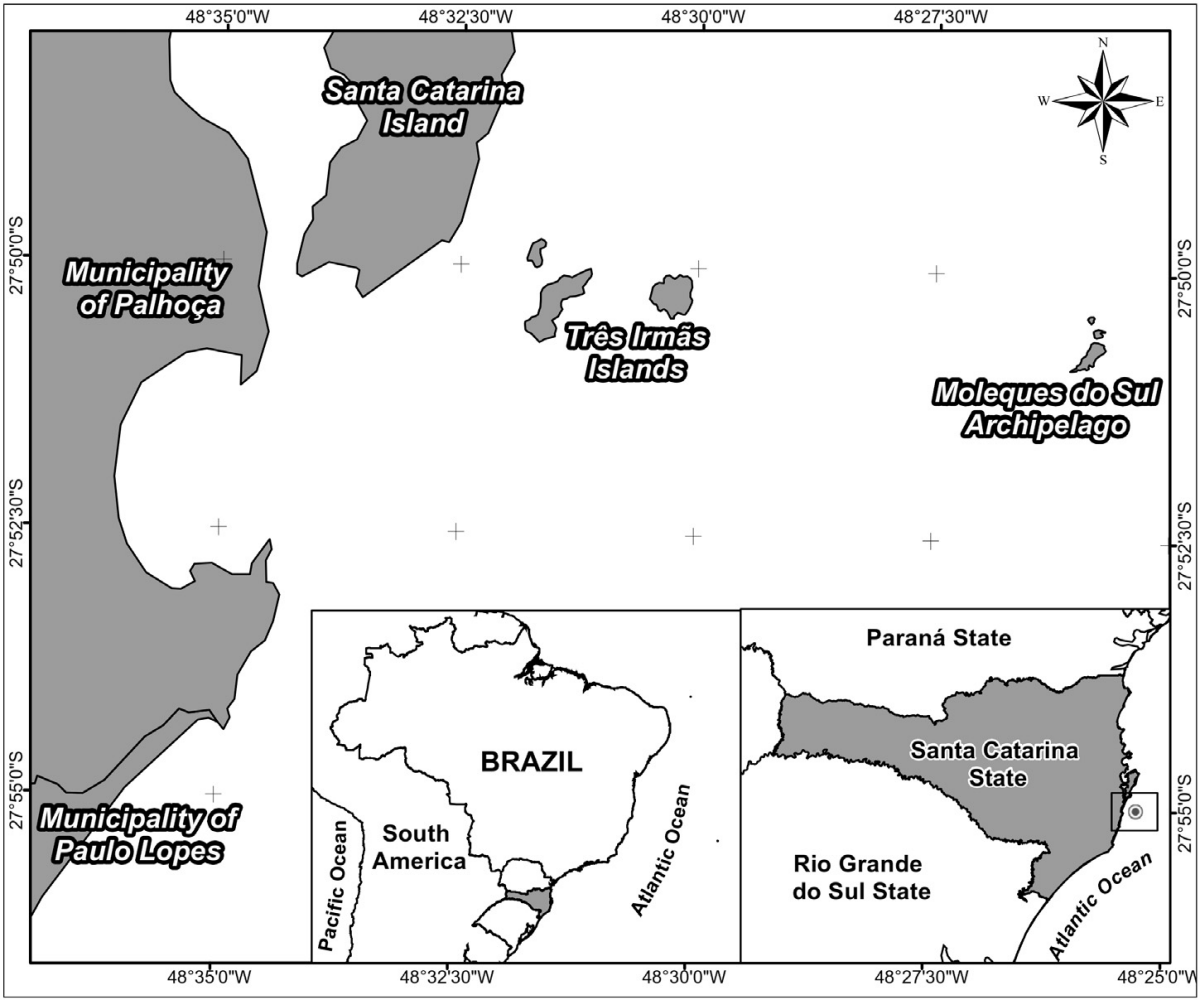
Location map of Moleques do Sul Archipelago.

The Moleques do Sul Archipelago (27°51′S; 48°26′W) consists of three oceanic islands. It is located 8 km from Santa Catarina Island and 14 km from the coast of Brazil, and is part of the Serra do Tabuleiro State Park in Santa Catarina State, Southern Brazil (Fig. 1). A detailed description of the area was provided by Salvador and Fernandez (2008).

*Cavia intermedia* were captured over 9 days in December, 2009, and for 4 days in February, 2010, using 34 traps baited with maize and placed in areas of high host density. Animals captured were numbered with ear-tags (Fish and small animal tag size 1, National Band and Tag Co., Newport, Kentucky, USA) and released at the place of capture. Techniques were approved by the Brazilian Federal Wildlife Agency (IBAMA) (license number # 033/07, process number # 02026.000394/2007-18) and are in accordance with guidelines published by the American Society of Mammalogists for use of wild mammals in research (Gannon and Sikes, 2007).

Lice were collected by brushing the hair coat on to a white tray, after rubbing cotton with ethyl ether on the host's body, and were then preserved in 70% ethanol. The sorting and counting of the lice was performed using a stereomicroscope. At least one sample containing several lice from each individual host was stored on permanent slides and identified according to Werneck (1942, 1948) and Emerson and Price (1975). Both the validity of specific names of lice and host were confirmed based on Price et al. (2003).

Chiggers were collected from the ears of the hosts by using forceps and they were stored in 70% alcohol. They were mounted in Hoyer's medium and examined on a light microscope with phase-contrast optics, according to Krantz and Walter (2009). They were identified according to Brennan and Goff (1977) and Brennan and Jones (1964), following the terminology of Goff et al. (1982). All mites and lice have been deposited at the Acari Collection of Instituto Butantan (IBSP130, IBSP11989, IBSP11990, IBSP11991).

Prevalence and abundance of lice were calculated according to Bush et al. (1997). Differences in abundance and prevalence among louse species were evaluated using a t-test and chi-square, respectively. Possible differences in parasite abundance (all species together or separately) between male and female hosts were evaluated with a t-test. Before performing the t-test, Levene's test was used to evaluate the homoscedasticity of the data. The parasitological parameters were not calculated for chiggers, because the collection of these parasites was not standardized.

## 3 Results and discussion

**Fig. 2:**
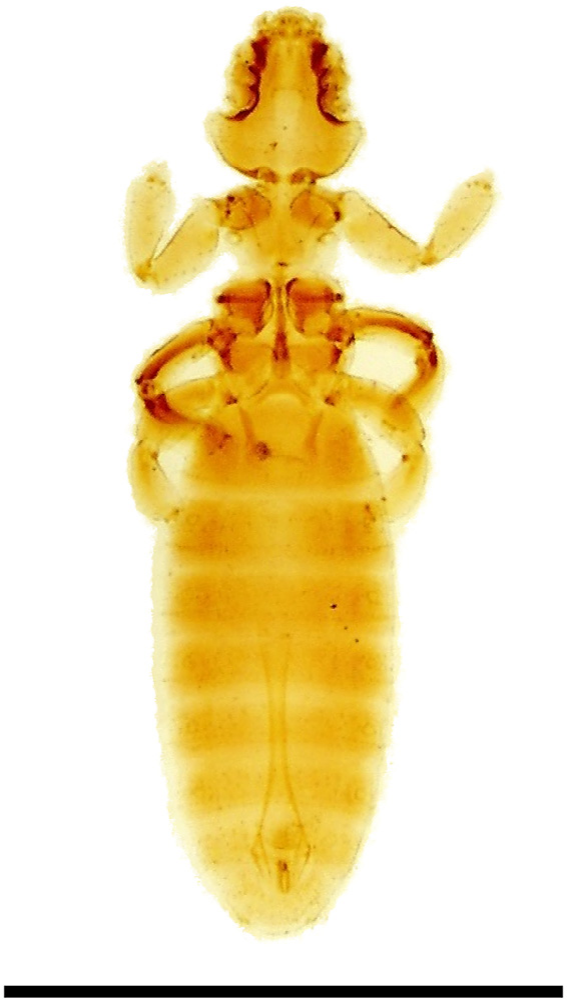
*Gliricola lindoiphoi* male collected on *Cavia intermedia.* The bar corresponds to 100 µm.

**Fig. 3:**
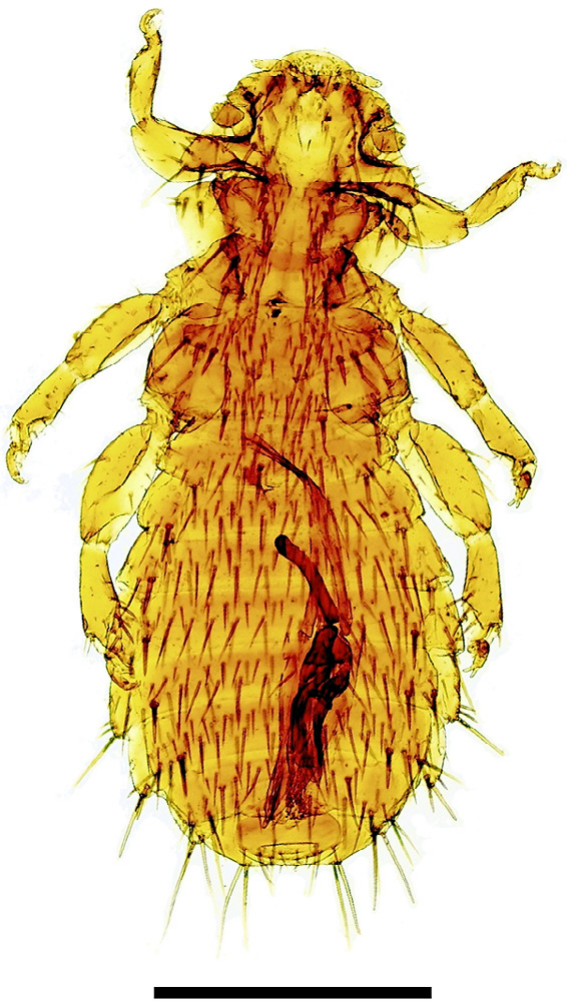
*Trimenopon hispidum* male collected on *Cavia intermedia.* The bar corresponds to 100 µm.

Twenty-seven *C. intermedia* (14 males and 13 females) were captured, which corresponds to approximately 65% of the total population as estimated by Salvador and Fernandez (2008). 1336 Mallophaga of two species were collected, *Gliricola lindolphoi* (Amblycera: Gyropidae) (Fig. 2) and *Trimenopon hispidum* (Amblycera: Trimenoponidae) (Fig. 3; Table 1). The morphological diagnosis of *G. lindolphoi* was based on: meso and methatorax fused into pterothorax; maxillary palpi 2-segmented; male genitalia with elongate and wide basal plate; straight parameres; genital sac with many sclerites; females presenting the longest terminal seta of the posterior margin shorter than the length of the last tergite. The morphological diagnosis of *T. hispidum* was based on: subtriangular head with straight lateral and posterior margins; pigmented eyes; presence of two claws on each of tarsi II–III and five pairs of abdominal spiracles.

**Fig. 4:**
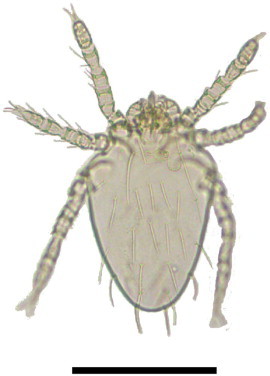
*Arisocerus hertigi* collected on *Cavia intermedia.* The bar corresponds to 200 µm.

Chiggers of two species, *Arisocerus hertigi* (Acari: Trombiculidae) (Fig. 4) and *Eutrombicula* sp. (Acari: Trombiculidae), were collected from the ears of all captured cavies. The morphological diagnosis of *A. hertigi* were based on: palpal tarsus with 7 branched setae; galeal seta nude; tibial claw trifurcate; 3 genualae I; a genuala II and III; a tibiala III and a mastitarsala III; palpal setae B/B/NNN; coxal setae 1.1.1; 2 pairs of sternal setae; PL>AL>AM, 20–22 dorsal setae; arranged 2H-6-6-(2–4)-2; 12–15 ventral setae; arranged 2st-2st-8-2-2; the sensilla are unilaterally expanded only one side and PL setae are long. The morphological diagnosis of *Eutrombicula* sp. were based on: palpal tibial claw bifurcate; cheliceral blade with tricuspid cap; palpal tarsus with 7 branched setae; a subterminala; and a tarsala; scutum roughly rectangular; wider than long; sensillae branched flageliform; five scutal setae; eyes 2/2, in a plate; leg segmentation 7-7-7; two or 3 genualae I; one genuala II and III; one tibiala III; 0–2 mastitibialae III; 1–3 mastitarsalae III.

Prevalence did not differ significantly among the two louse species and was high for both (χ 2 = 0.18, df = 2, p = 0.915). The mean abundance was 49.5 (±39.1) parasites/host. Abundance of *T. hispidum* was greater than that of *G. lindolphoi* (Table 1) (t = 3.54, df = 26, p = 0.001). Host sex did not affect mean abundance (t = −0.5, df = 25, p = 0.62) or the abundance of either louse species individually (*T. hispidum*: t = −1.033, df = 25, p = 0.311; *G. lindolphoi*: t = −0.049, df = 25, p = 0.961).

This work shows, for the first time, the occurrence of two chewing louse species, *T. hispidum* and *G. lindolphoi*, and two trombiculid mites, *A. hertigi* and *Eutrombicula* sp., associated with *C. intermedia*, a rodent species highly endangered and endemic to an island in southern Brazil. Approximately 50 species of ectoparasites have been reported for the genus *Cavia* (Table 2), but in the current study only a few were found on *C. intermedia*. This low number of parasite species is expected for island mammals, as reported for *C. fulgida* (Guitton et al., 1986) and *C. porcelllus* (Linardi et al., 1991). The structure of parasites assembly on islands is strongly related to the low richness of free-living species typical of these environments (MacArthur and Wilson, 1967). This occurs because the parasites depend on the hosts to disperse to these sites and also to establish, especially for those who require more than one species of host to complete its life cycle (Magnanou and Morand, 2006).

Until now, *C. aperea, C. fulgida, C. a. pamparum* and *C. porcellus* were reported as hosts of *T. hispidum*. This louse species is common in these hosts, but *G. lindolphi* is known only from *C. aperea* (Table 2), and is rare, being reported here for only the sixth time (Emerson and Price, 1975; Cardozo-de-Almeida et al., 2003; Krüger, 2006).

*Arisocerus hertigi* was originally described in rodents (Dasyproctidae) and marsupials from Sommerfield, Paraguay (Didelphidae) (Brennan and Jones, 1964). Subsequently, this species was also found parasitizing marsupials (Didelphidae) in the Federal District, Brazil (Goff and Gettinger, 1989). Therefore, this work reports the first record of this species in Caviidae.

The genus *Eutrombicula*, the most important in terms of human and animal health in the Neotropical Region, is composed of about 80 species (Brennan and Reed, 1974; Stekol′Nikov and González-Acuña, 2010). According to Daniel and Stekol′Nikov (2004) many species of this genus were identified “by default”. Thus, the correct identification of the material from *C. intermedia* will be possible only after a taxonomic revision of the genus.

We observed 100% prevalence and an abundance of 33 parasites/host for *T. hispidum* on *C. intermedia.* These values are slightly higher than those found in two other studies of *Cavia* spp., except for a location where the estimated abundance was much lower. Valim et al. (2004) estimated a prevalence of 100% and an abundance of 4.8 parasite/host and a prevalence of 90% and abundance of 29.1 parasites/host for *T. hispidum* on *C. porcellus* in Duque de Caxias and Silva Jardim in Rio de Janeiro State, Brazil, respectively. Krüger (2006) reported a prevalence of 97% and abundance of 23 parasites/host for *T. hispidum* on *C. aperea* from Pelotas, Rio Grande do Sul State, Brazil. While the prevalence of *G. lindolphoi* in this study (89%) is much higher than previously reported by Krüger (2006) (48%), the abundance (17.4 parasites/host) is similar to that reported by this author on *C. aperea* (16 parasites/host).

The differences between these studies and the results reported here are possibly related to high population densities of the *C. intermedia*, as stated by Salvador and Fernandez (2008). Since the transmission of lice occurs through direct contact between hosts, it is expected that parasite prevalence and abundance are positively related to population density of the host (Stanko et al., 2002; Magnanou and Morand, 2006). Although some studies reported differences in abundance between host genders perhaps linked to host behavior (Soliman et al., 2001; Moore and Wilson, 2002) or hormonal (Dlugosz et al., 2014) differences, there were no significant differences for *C. intermedia*.

## 4 Conclusion

The results presented here do not provide a complete picture of the relationship between *C. intermedia* and its ectoparasites, because this type of interaction is dynamic and changes with time (Linardi and Guimarães, 2000). However, we found some evidence for the effects of insularity on parasitism: a parasite fauna of low richness; parasitological parameters different from those found in studies on other *Cavia* spp. on the mainland of Brazil; the presence of generalist species (trombiculid mites); and direct life cycle species (lice) (Magnanou and Morand, 2006). Parasitological studies of *Cavia* spp., especially of *C. magna*, may help to clarify the interaction of *C. intemedia* and its ectoparasites.

Although the results presented here are not conclusive about the relationship between *C. intermedia* and ectoparasites, this low species richness found might be reflected in a low level of investment by the hosts in the basal immune defense, since investments in white blood cell production by mammals are influenced by the diversity of parasites in the environment (Bordes and Morand, 2009). Additionally, considering that it might result in host vulnerability to other parasites that might be introduced through exotic or migratory host species, the monitoring of *C. intermedia*, including parasitological and immunological assessments, is recommended as a key component of conservation efforts.

## Figures and Tables

**Table 1:** Abundance and prevalence of louse species associated with *Cavia intermedia* at Moleques do Sul Island, Santa Catarina, Brazil. Legend: ♂ = male adults; ♀ = female adults and Ny = nymphs.

Louse species	Prevalence (%)	Abundance (parasites/host)	Sex and developmental stage			Total
			♂	♀	Ny	
*Trimenopon hispidum*	100	32.0	299	243	323	865
*Gliricola lindolphoi*	89	17.4	96	113	262	471
Total	_	49.5	395	356	585	1336

**Table 2:** Checklist of arthropods parasites from *Cavia* spp.

Ectoparasites	Hosts				
	*Cavia aperea*	*Cavia aperea pamparum*	*Cavia porcellus*	*Cavia fulgida*	*Cavia intemedia*
Phthiraptera					
* Gliricola brasiliensis*	_	_	Cardozo-de-Almeida et al. (2003)	_	_
* Gliricola lindolphoi*	Emerson and Price (1975); Cardozo-de-Almeida et al. (2003); Krüger (2006)	_	Emerson and Price (1975)	_	This study
* Gliricola porcelli*	Emerson and Price (1975); Dittmar (2002); Krüger (2006)	Castro et al. (1987)	Emerson and Price (1975); Linardi et al. (1991); Dittmar (2000); Cruz et al. (2003); Valim et al. (2004)	Guitton et al. (1986)	_
* Gliricola spinosus*	Cardozo-de-Almeida et al. (2003)	_	_	_	_
* Gyropus ovalis*	Emerson and Price (1975); Krüger (2006)	Emerson and Price (1975); Castro et al. (1987)	Emerson and Price (1975); Cruz et al. (2003); Valim et al. (2004)	Emerson and Price (1975); Guitton et al. (1986)	_
* Polyplax spinulosa*	Dittmar (2002); Cruz et al. (2003)	_	_	_	_
* Pterophthirus alata*	Dittmar (2002)	Castro et al. (1987)	_	_	_
* Pterophthirus imitans*	_	Castro et al. (1987)	_	_	_
* Trimenopon hispidum*	Emerson and Price (1975); Cicchino and Castro (1984); Krüger (2006)	Cicchino and Castro (1984); Castro et al. (1987)	Emerson and Price (1975); Cicchino and Castro (1984); Dittmar (2000); Cruz et al. (2003); Valim et al. (2004)	Emerson and Price (1975); Cicchino and Castro (1984); Guitton et al. (1986)	This study
Siphonaptera					
* Adoratopsylla intermedia intermedia*	Linardi and Guimarães (2000)	_	_	_	_
* Ctenocephalides felis felis*	_	_	Linardi and Guimarães (2000); Cruz et al. (2003)	_	_
* Echidnophaga galinacea*	_	_	Cruz et al. (2003)	_	_
* Leptopsylla segnis*	Dittmar (2002); Cruz et al. (2003)	_	_	_	_
* Polygenis atopus*	Dittmar (2002); Cruz et al. (2003)	_	_	_	_
* Polygenis axius*	Krüger et al. (2010)	_	_	_	_
* Polygenis bohlsi jordani*	Linardi and Guimarães (2000)	_	Linardi and Guimarães (2000)	_	_
* Polygenis frustratus*	Linardi and Guimarães (2000)	_	_	_	_
* Polygenis platensis*	_	Castro et al. (1987)	_	_	_
* Polygenis rimatus*	_	Castro et al. (1987)	_	_	_
* Pulex simulans*	_	_	Dittmar (2000)	_	_
* Pulex* sp.	_	_	Cruz et al. (2003)	_	_
* Tiamastus cavicola*	Linardi and Guimarães (2000); Dittmar (2002)	Castro et al. (1987)	_	_	_
* Xenopsylla cheopis*	Linardi and Guimarães (2000)	_	Cruz et al. (2003)	_	_
Acari					
* Amblyomma tigrinum*	_	Castro et al. (1987)	_	_	_
* Androlaelaps fahrenholzi*	_	Castro et al. (1987)	_	_	_
* Arisocerus hertigi*	_	_	_	_	This study
* Cavilaelaps bresslaui*	_	Castro et al. (1987)	_	_	_
* Chirodiscoides caviae*	_	_	Valim et al. (2004)	_	_
* Chirodiscoides caviae*	_	_	Cruz et al. (2003)	_	_
* Dermanyssus gallinae*	_	_	Cruz et al. (2003)	_	_
* Eubrachylaelaps batatas*	_	_	Cruz et al. (2003)	_	_
* Eubrachylaelaps rotundus*	_	Castro et al. (1987)	_	_	_
* Euchoengastia pazca*	_	Castro et al. (1987)	_	_	_
* Eulaelaps stabularis*	_	Castro et al. (1987)	_	_	_
* Eutrombicula alfreddugesi*	_	Castro et al. (1987)	_	_	_
* Eutrombicula bruyanti*	Dittmar (2002); Cruz et al. (2003)	_	_	_	_
* Eutrombicula* sp.	_	_	_	_	This study
* Gigantolaelaps mattogrossensis*	_	Castro et al. (1987)	_	_	_
* Myobia musculi*	Dittmar (2002); Cruz et al. (2003)	_	_	_	_
* Myocoptes musculinus*	Cruz et al. (2003)	_	Cruz et al. (2003)	_	_
* Mysolaelaps microspinosus*	_	Castro et al. (1987)	_	_	_
* Neolaelaps bispinosus*	_	_	_	Guitton et al. (1986)	_
* Neoparalaelaps bispinosus*	_	Castro et al. (1987)	_	_	_
* Notoedres muris*	_	_	Cruz et al. (2003)	_	_
* Ornithonyssus bacoti*	_	Castro et al. (1987)	Cruz et al. (2003)	_	_
* Ornithonyssus brasiliensis.*	_	_	Evans et al. (2000)	_	_
* Ornithonyssus lutzi*	_	_	Bastos (2008)	_	_
* Ornithonyssus monteiroi*	Bastos (2008)	_	_	_	_
* Ornithonyssus* spp.	_	_	Dittmar (2000)	_	_
* Ornithonyssus vitzthumi*	Bastos (2008)	_	_	_	_
* Ornithonyssus wenwki*	_	_	Cruz et al. (2003)	_	_
* Ornithonyssus wernecki*	Cruz et al. (2003)	_	_	_	_
